# ICP4-Associated Activation of Rap1b Facilitates Herpes Simplex Virus Type I (HSV-1) Infection in Human Corneal Epithelial Cells

**DOI:** 10.3390/v15071457

**Published:** 2023-06-27

**Authors:** Beibei Zhang, Juntao Ding, Zhenghai Ma

**Affiliations:** Xinjiang Key Laboratory of Biological Resources and Genetic Engineering, College of Life Science and Technology, Xinjiang University, Urumqi 830046, China; zhangbeibei2022@xju.edu.cn (B.Z.); dingjuntao2004@126.com (J.D.)

**Keywords:** RAS-related protein 1b, herpes simplex virus type I, protein kinase A, ICP4, signal pathway, cytoskeleton

## Abstract

The strong contribution of RAS-related protein 1b (Rap1b) to cytoskeleton remodeling determines intracellular and extracellular physiological activities, including the successful infection of viruses in permissive cells, but its role in the HSV-1 life cycle is still unclear. Here, we demonstrated that the HSV-1 immediate early (IE) gene ICP4 inhibits protein kinase A (PKA) phosphorylation to induce Rap1b-activation-mediated viral infection. Rap1b activation and membrane enrichment begin at the early stage of HSV-1 infection and remain active during the proliferation period of the virus. Treating the cells with Rap1b small interfering RNA (siRNA) showed a dose-dependent decrease in viral infection levels, but no dose-dependent increase was observed after Rap1b overexpression. Further investigation indicated that the suppression of Rap1b activation derives from phosphorylated PKA and Rap1b mutants with partial or complete prenylation instead of phosphorylation, which promoted viral infection in a dose-dependent manner. Furthermore, the PKA agonist Forskolin disturbed Rap1b activation in a dose-dependent manner, accompanied by a decreasing trend in viral infection. Moreover, the HSV-1 IE gene ICP4 induced PKA dephosphorylation, leading to continuous Rap1b activation, followed by cytoskeleton rearrangement induced by cell division control protein 42 (CDC42) and Ras-related C3 botulinum toxin substrate 1 (RAC1). These further stimulated membrane-triggered physiological processes favoring virus infection. Altogether, we show the significance of Rap1b during HSV-1 infection and uncover the viral infection mechanism determined by the posttranslational regulation of the viral ICP4 gene and Rap1b host protein.

## 1. Introduction

Herpes simplex virus type I (HSV-1) is a double-stranded DNA envelope virus of the α Herpesvirus subfamily and a member of the herpes simplex virus genus. HSV-1 is an exclusively human pathogen, prevalent worldwide, with an extremely high infection rate in the population [1]. HSV-1 has significant neural tissue tropism, initially invading facial epithelial cells but then transferring to the trigeminal nerve through adjacent sensory neurons, where it establishes lifelong latent infection with periodic reactivation, leading to subclinical or mild clinical symptoms characterized by the development of blisters, sores, ulcers, or keratitis in the primary infected areas [2,3,4]. However, these repeated injuries can also induce serious diseases, such as herpetic encephalitis (HE; 0.003%) and blindness (0.013%) [5,6]. Of note, the mortality rate in patients with HE can still reach 30% even after treatment (70% without treatment), accompanied by a high incidence of neurological sequelae [7]. Although the antiviral drug acyclovir (ACV) can improve the prognosis and treatment of HSV-1 infection through topical application, injection, or oral administration, ACV is ineffective after the virus infiltrates the brain [8,9]. Therefore, controlling the infection in the primary sites is crucial to avoiding viral persistence and the resulting severe diseases. 

The HSV-1 genome size is about 152 kb with more than 80 open reading frames (ORFs), including immediate early (IE, or α), early (E, or β), and late (L, or γ) genes, encoding at least 80 multifunctional proteins [10,11]. Interestingly, five infected cell polypeptides (ICP) of IE genes—ICP0, ICP4, ICP22, ICP27, and ICP47—are expressed first to initiate the replication program through collaboration within the genome and containment at different infection stages during the lytic life cycle to maximize the efficiency of virus replication [10,12]. Among them, the translation product of ICP4 was identified as a phosphorylated protein with a molecular weight of ~180 kDa, consisting of an N- and C-terminal catalytic domain, a DNA binding domain, and linkers, indicating its multiple functions [13]. ICP4 was originally defined as a trans-activator of E and L gene expression based on its regulation of promoter activity under different temperatures [14]. The absence of ICP4 severely impairs viral replication by stalling the transcription and translation of E and L viral genes [15]. Chaturvedi et al. showed that ICP4 achieved auto-repression activity after extrinsic stimuli through its transfer of intracellular dynamics rather than an increase in the amount of ICP4 protein or mRNA expression [16]. Additionally, ICP4 can act as an activator and repressor of host RNA polymerase II during infection, which may facilitate replication or inhibit antiviral responses [17]. ICP4 can also promote ataxia telangiectasia [18], the formation of viral replication compartments [19], the stability of host factor-mediated transcriptional catalytic activity [20], and microtubule-mediated nuclear transfer [21] to favor replication conditions and environments. 

As a member of the Ras oncogene family, Rap1b can bind to guanosine triphosphate (GTP) or guanosine diphosphate (GDP) during the transition process between activated Rap1b-GTP and inactivated Rap1b-GDP states, which is regulated by the guanylate exchange factor (GEF) and GTPase activating protein (GAP), respectively [22]. Uncontrolled Rap1b activation is often associated with the occurrence of tumors, especially highly metastatic malignant ones [23], in which the process of Rap1b-induced regulation of the cytoskeleton is significant [24]. Nevertheless, cytoskeleton rearrangement occurs during early HSV-1 viral entry, intracellular trafficking, and release and can be hijacked by the viruses to facilitate viral replications such as those of porcine hemagglutinating encephalomyelitis virus (PHEV) [25], hepatitis E virus (HEV) [26], and severe acute respiratory syndrome coronavirus 2 (SARS-CoV-2) [27,28,29]. However, the impact of Rap1b in regulating cell membranes during viral replication has rarely been assessed aside from its role in the proliferation and migration of hepatitis-B-infected hepatocytes [30]. Interestingly, membrane enrichment of activated Rap1b initiates a signaling cascade with multiple host factors that accelerate high-efficiency membrane remodeling and boost early HEV infection [26]. Mechanistically, the two consecutive serines (aa179 and aa180) in the polybasic binding region (PBR) of Rap1b are prone to phosphorylation, while the following cysteine (aa181) keeps the most limited prenylation and activation under the septal inhibition from phosphorylation to achieve the recurrent cycle of the membrane and cytoplasmic transfer for the most favorable balance in terms of function and energy saving during different physiological processes, including viral infection [22]. Previous studies have shown that crosstalk pathways led by downstream CDC42 play a decisive role in membrane remodeling during viral infection in the Rap1b-triggered cascade signaling pathway, which significantly differs between enveloped and non-enveloped viruses [31]. The Rho GTPase family proteins, the ras homolog family member A (RhoA), CDC42, and RAC1, are widely defined as effectors involved in cytoskeleton regulation [32], and numerous studies have found that the Rho GTPase family is involved in targeting the cytoskeleton in the process of virus infection, such as dengue virus (DENV) [33], African swine fever virus (ASFV) [34], pseudorabies virus (PRV) [35], etc. [36]. Coincidentally, all of these three protein expressions as well as their activation were up-regulated during the early infection of HSV-1 in human trabecular meshwork cells, and siRNA and drugs that blocked activation showed significant inhibition of cytoskeleton rearrangement and virus infection [37]. Cofilin, which controls actin dynamics and membrane remodeling directly (lamellipodia, filopodia, and stress fibers), has also been associated with HSV-1 during early infection [38,39]. Altogether, these reports hint at a potential relationship between Rap1b, the cytoskeleton, and viral infection.

Due to the diversified functions of ICP4, including the cytoskeleton’s regulation ability, ICP4 may be associated with the HSV-1 infection process involving the cell membrane [16,17,19,20]. That said, its mechanism is unknown. Interestingly, Rap1b, characterized by its spatiotemporal expression manner (dynamic transfer of cytoplasm and intima) during activation state transition, has been firmly identified as being related to normal physiological processes, tumorigenesis, and viral infection resulting from its regulation of membrane dynamic changes [22,30]. Meanwhile, epithelial cells, as the primary site of HSV-1 infection, are crucial for the establishment of infection [40], and blocking the primary infection at this site may also be the most effective way to prevent infection. Therefore, a hypothesis about how Rap1b responds to ICP4 to regulate membrane-involved infection emerged spontaneously; this paper presents evidence for the proposed model on HSV-1-infected human corneal epithelial cells. The results indicate that HSV-1 infection promoted the membrane enrichment of Rap1b, leading to the activation of CDC42 and RAC1, as well as the frequent conversion of Cofilin between its activated (dephosphorylation) and deactivated (phosphorylation) states [41], which in turn promoted membrane remodeling dominated by F-actin, thus promptly infecting the virus. After screening five HSV-1 IE genes, we determined that ICP4 is likely responsible for the activation of Rap1b, and this is achieved by inhibiting the activation of the phosphorylation restriction factor PKA [22], allowing for effective viral infection. These findings will provide a novel therapeutic avenue to block HSV-1 persistence and recurring symptoms, enabling the prevention of neurological disease from primary infection sites.

## 2. Materials and Methods

### 2.1. Cells, Virus, Plasmids, Chemicals, and Antibodies

Human corneal epithelial (HCE) and Vero cells used in this study were obtained from the American Type Culture Collection (ATCC), while HCE cells were maintained in the M199 medium (Gibco, Carlsbad, CA, USA) with 10% fetal bovine serum (FBS; Gibco) supplemented with 100 U/mL penicillin and 100 mg/mL streptomycin (Thermo Fisher Scientific; Waltham, MA, USA), and Vero cells were grown in the DMEM (Gibco) medium with the same amount of supplements of FBS and antibiotics; both of them were cultured at 37 °C in a cell incubator with 5% CO2. Human alphaherpesvirus 1 strain 17 (HSV-1 17; GenBank number: NC_001806.2) was isolated from the Regional Virus Laboratory, Ruchill Hospital, Glasgow, United Kingdom, and was kindly gifted from Prof. Bernard Roizman (Department of Microbiology, University of Chicago), propagated in Vero cells, and titrated using plaque assay. The backbone plasmids pcDNA3 and pCMV were obtained from Beyotime Biotechnology (Beijing, China). 

For the chemical agents, Forskolin, H-89, and CytoD were purchased from Selleck Chemicals (Houston, TX, USA) and diluted in dimethyl sulfoxide (DMSO) at working concentrations. FITC-labeled phalloidin was obtained from Solarbio Life Science (Beijing, China). Antibodies against Rap1b, GAPDH, Na+/K+ ATPase, PKA, CDC42, Cofilin, and RAC1 were obtained from ProteinTech (Wuhan, China). Antibodies against p-PKA and p-Cofilin were obtained from Cell Signaling Technology (Boston, MA, USA). Antibodies against gD and ICP4 were obtained from Abcam (Cambridge, United Kingdom). Cy5-conjugated goat anti-mouse IgG (H+L), Alexa Fluor^®^ 488-conjugated goat anti-mouse IgG (H+L), HRP-conjugated goat anti-mouse IgG (H+L), and HRP-conjugated goat anti-rabbit IgG (H+L) were obtained from Jackson ImmunoResearch (West Grove, PA, USA), Abcam, and TransGen Biotech (Beijing, China), respectively.

### 2.2. Construction and Transfection of Recombinant Plasmids 

To understand Rap1b activation and ICP4-positive feedback on Rap1b activation during HSV-1 infection of HCE cells, eukaryotic transient expression plasmids were constructed using pcDNA3 and pCMV as the backbone plasmids, respectively. Specifically, RNA extraction was performed using RNAiso Plus (TaKaRa, Dalian, China) from HCE cells according to the manufacturer’s instructions, followed by a reverse transcription process with the PrimeScript RT reagent kit (TaKaRa) to obtain cDNA; virus DNA was obtained using a DNA Viral Genome Extraction Kit (Solarbio Life Science), also according to the manufacturer’s instructions. Wild-type Rap1b, its mutants, and five HSV IE genes were amplified with PrimeSTAR^®^ Max DNA Polymerase (TaKaRa) according to the manufacturer’s instructions, using the abovementioned cDNA and viral DNA as templates. Afterward, PCR products were recovered with an EasyPure^®^ Quick Gel Extraction Kit (TransGen Biotech, Beijing, China) after separation using 1.5% agarose (Beyotime Biotechnology) gel electrophoresis. The obtained DNA fragments and vectors were subjected to double enzyme digestion with *Eco*R I and *Xho* I, or *Eco*R I and *Xba* I (TaKaRa), respectively, and purified with the same EasyPure^®^ Quick Gel Extraction Kit above. Finally, digested fragments and vectors were ligated at 16 °C for 30 min using a DNA Ligation Kit (TaKaRa) to obtain the recombinant plasmids. After bacterial transformation screening (DH5α cells) and sequencing, plasmids were expanded with a PureLink™ Expi Endotoxin-free Massive Plasmid Purification Kit (Thermo Fisher Scientific). All primer pairs used are listed in Table 1.

HCE cells were seeded in the 6-well and 24-well plates (Thermo Fisher Scientific) at a density of 2 × 10^5^/mL and 1 × 10^5^/mL, respectively, and cultured overnight to reach a ~70% share of the bottom area. Cells were transfected with recombinant or backbone plasmids with Lipo8000TM transfection reagent (Beyotime Biotechnology) according to the manufacturer’s instructions and maintained for 24 h. Cells were then collected for the detection of target protein expression or infected with a virus and harvested for the evaluation of target protein expression and viral titer using Western blotting, TCID_50_, or IFA assays.

### 2.3. Virus Infection

HCE cells were seeded in the 6-well and 24-well plates (Thermo Fisher Scientific) at a density of 2 × 10^5^/mL and 1 × 10^5^/mL, respectively, and cultured overnight. Cells were prechilled on ice for 10 min and infected with HSV-1 at an MOI of 2 at different time points. To confirm Rap1b activation during HSV-1 infection, cells were collected directly at the indicated time points for Rap1b activation and membrane transfer assays using Western blotting and IFA. For experiments involving siRNA or overexpression, cells were subjected to the same viral infection steps as above after transfection with indicated amounts or concentrations, and viral titer and target protein expression were detected using Western blotting, TCID_50_, or IFA assays. For experiments involving drug application, cells were pretreated with the indicated concentrations of drugs or DMSO at 37 °C for 1 h, followed by a similar viral infection protocol described above in the presence of inhibitors or agonists. Viral titer, target protein expression, and membrane remodeling were detected using Western blotting, TCID_50_, or IFA assays at the indicated time points.

### 2.4. Protein Extraction and Expression Level Evaluation 

Cell samples treated as above were washed twice with ice-cold PBS and harvested using centrifugation at 1500× *g* for 5 min at 4 °C. The protein content was extracted and quantified with NP40 lysis buffer (Beyotime Biotechnology) and a Pierce BCA Protein assay kit (Thermo Fisher Scientific), respectively, according to the manufacturer’s instructions, and then further analyzed using sodium dodecyl sulfate-polyacrylamide gel electrophoresis (SDS-PAGE) and Western blotting to evaluate the expression of different target proteins [26,31]. For the evaluation of Rho family proteins with GTP loading [42], active forms of Rap1b (Rap1b-GTP), CDC42 (CDC42-GTP), and RAC1 (RAC1-GTP) were enriched from treated cells using an Active Rap1b Detection Kit and an Active CDC42 and RAC1 Detection Kit (Cell Signaling Technology), respectively, according to the manufacturer’s instructions, and then analyzed using Western blotting. The housekeeper proteins GAPDH and Na+/K+ ATPase were selected as internal references for total and membrane protein fractions, respectively, as loading controls. 

### 2.5. Immunofluorescence Assay (IFA)

To confirm the correlation between the virus, Rap1b, and/or the cytoskeleton, or their relationship after treatments with chemicals or plasmids in HSV-1-infected cells, target-protein-specific primary antibodies, fluorescent-labeled secondary antibodies, or a fluorescently labeled chemical (phalloidin) were applied in IFA as previously described [26,31]. Cell climbing sheets and cell plates were observed with a Leica SP8 confocal system (Leica Microsystems, Wetzlar, Germany). All images were captured and processed using Leica Application Suite X (Leica Microsystems).

### 2.6. Small Interfering RNA (siRNA) Interference

HCE cells were seeded in a 12-well plate (Thermo Fisher Scientific) at a density of 2 × 10^5^/mL and cultured overnight to a confluence of 50~60% bottom area. Cells were then transfected with Rap1b or negative control siRNAs (GenePharma Co., Ltd.; Shanghai, China) with Lipofectamine RNAiMAX reagent (Thermo Fisher Scientific) according to the manufacturer’s instructions. After 24 h of incubation, cells were infected with the HSV-1 virus, and the expression of target proteins and viral titers were measured using Western blotting, TCID_50_, or IFA assays. The siRNA sequences used are listed in Table 2.

### 2.7. Virus Titration

HSV-1 virus from infected cells was collected using three rounds of repeat freeze–thaw cycles of –80 °C and 37 °C, and viral particles were harvested using centrifugation for titration as previously described [43]. Briefly, Vero cells were seeded overnight in a 96-well plate at a density of 1 × 10^4^/mL. Pelleted viral particles were serially diluted 10 times from 1:10 in six replicates of each dilution to observe positive cells with a cytopathic effect (CPE). The virus titer was calculated using a 50% tissue culture infectious dose (TCID_50_) based on the Reed–Muench method [44].

### 2.8. Cell Viability Analysis

As previously described, cytotoxicity in HCE cells was determined with the CCK-8 Kit (Beyotime Biotechnology) [26]. Briefly, cells were seeded in a 96-well plate at a density of 1.5 × 10^4^/mL overnight, treated as described above, and then incubated with a CCK-8 solution for 2 h. The absorbance at 450 nm was measured with an Epoch-BioTek Microplate Reader (Winooski, VT, USA). Mock-treated and DMSO-treated cells served as the blank and negative controls, respectively. 

### 2.9. Statistical Analysis

Data analysis and visualization were performed with GraphPad Prism version 8.0 software (GraphPad Software, San Diego, CA, USA). Data are presented as mean ± SD. Statistical significance between two or more groups was determined using the Student’s *t*-test or analysis of variance (ANOVA), respectively. A two-tailed *p* value < 0.05 with a 95% confidence interval was considered statistically significant.

## 3. Results

### 3.1. HSV-1 Infection Induces Rap1b Activation and Membrane Distribution

**Figure 1:**
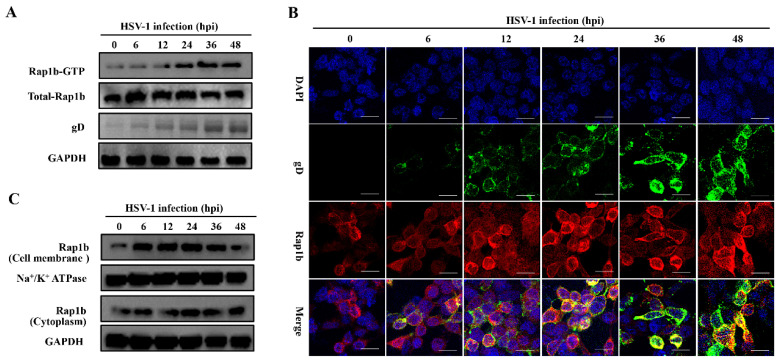
Activation of Rap1b and membrane transfer after HSV-1 infection. (**A**) Rap1b activation assay after HSV-1 infection. HCE cells were infected with HSV-1 (MOI = 2) and lysed at indicated time points to detect viral gD, total Rap1b, and Rap1b-GTP proteins using Western blotting. GAPDH served as an internal loading control. (**B**,**C**) HSV-1 infection induced dynamic transfer of Rap1b between membrane and cytoplasm. HCE cells with HSV-1 (MOI = 2) infection were harvested at indicated time points, followed by fixation or cell component separation, for visual observation and evaluation of Rap1b using IFA and Western blotting, respectively. GAPDH served as an internal loading control. Scale bar: 20 μm.

By monitoring the Rap1b activation mode during HSV-1 infection, we found that total and activated Rap1b increased from an early stage with similar growth and decline trends (Figure 1A). Upon HSV-1 infection, the total amount of Rap1b protein was significantly up-regulated, peaking at 6 hpi and slightly decreasing at 12 hpi to levels still significantly elevated compared to the uninfected control. Rap1b activation (Rap1b-GTP) also increased at 6 hpi and continued to increase to a peak at 36 hpi and a plateau at 48 hpi. This difference between total protein up-regulation and activation dynamics suggests posttranslational modifications. We next assessed Rap1b activation and cellular localization using confocal microscopy. Rap1b is gradually recruited to the cell membrane after infection, peaking at 24 hpi (Figure 1B), which is consistent with the Rap1b activation dynamics on the cell membrane and in the cytoplasm (Figure 1C).

### 3.2. Rap1b Is Closely Associated with HSV-1 Infection 

**Figure 2:**
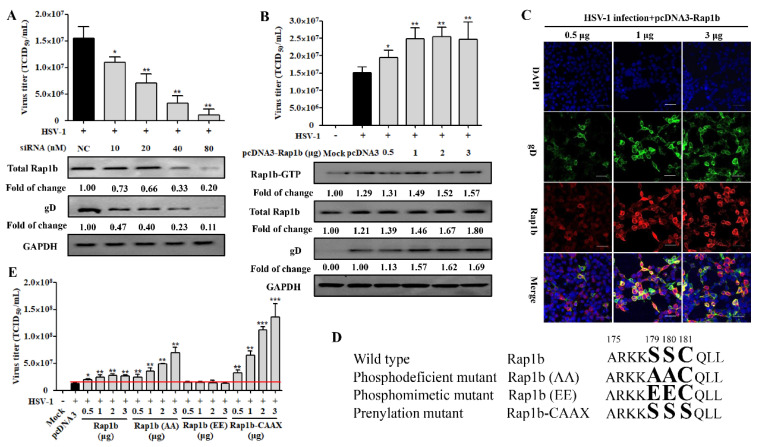
Rap1b is essential for HSV-1 infection, and its activation may be regulated using posttranslational modification. (**A**) siRNA knockdown of Rap1b impaired Rap1b activation and HSV-1 infection in a dose-dependent manner. HCE cells were transfected with different concentrations of Rap1b-specific siRNA (siRap1b) or negative control (NC) siRNA (80 nM); 24 h after transfection, cells were infected with HSV-1 (MOI = 2) for another 24 h. Viral titers were measured using TCID_50_, and the detection of viral gD and total Rap1b proteins was detected using Western blotting. GAPDH was used as an internal loading control. Mock-treated cells served as a blank control. Data represent the results from three independent experiments in duplicate (Mean ± SD, * *p* < 0.05, ** *p* < 0.01). (**B**) Transient transfection of Rap1b plasmid failed to promote Rap1b activation and virus infection in a dose-dependent manner. HCE cells were transfected with different amounts of recombinant Rap1b plasmid (pcDNA3-Rap1b) or control plasmid (pcDNA3, 3 μg) for 24 h and then infected with HSV-1 (MOI = 2) for another 24 h. Viral titers were measured using TCID_50_; viral gD and total Rap1b proteins were detected using Western blotting. GAPDH was used as an internal loading control. Mock-treated cells served as a blank control. HSV-1-infected cells treated with 0.5, 1, and 3 μg of pcDNA3-Rap1b were fixed for IFA of viral gD and Rap1b (**C**). Data represent the results from three independent experiments in triplicate (Mean ± SD, * *p* < 0.05, ** *p* < 0.01, *** *p* < 0.001). Scale bar: 80 μm (**D**) Schematic diagram of wild-type Rap1b and mutants. The PBR region at the carboxyl-terminal end of Rap1b with phosphorylation (aa179 and aa180) and prenylation (aa181) sites was selected for directed nucleotide mutagenesis with a primer-derived method for the construction of transient expression plasmid with pcDNA3 as the backbone vector. Enlarged and bold amino acids represent the schematic sites of Rap1b wild-type and PBR mutation mimetics (**E**) Differences in virus infection after transfection of wild-type Rap1b and mutants. The HCE cells were transfected with different amounts of the recombinant plasmids Rap1b, Rap1b (AA), Rap1b (EE), or Rap1b-CAAX for 24 h continuous cultivation of treated cells, then infected with HSV-1 (MOI = 2) for another 24 h. Finally, cells from indicated groups were collected for viral titration using TCID_50_. Mock-treated cells served as the blank control. Data represent the results from three independent experiments in triplicate (Mean ± SD, * *p* < 0.05, ** *p* < 0.01, *** *p* < 0.001). The red line represents the baseline level of virus titer in the pcDNA3 transfection group after viral infection.

Having established a correlation between HSV-1 infection and Rap1b expression, we next assessed whether Rap1b inhibition with siRNA could impair viral infection. As expected, viral infection was inhibited with siRNA in a dose-dependent manner. After the knockdown of Rap1b expression (≈80%), viral infection was reduced by 90% (Figure 2A). Viral titers were rescued via Rap1b plasmid transient overexpression but without a clear dose–response phenotype, even though the total Rap1b protein increased significantly during virus infection (Figure 2B,C). In contrast, the changing trend synchronous with total Rap1b is not captured at Rap1b-GTP assessment but is similar to the results of virus titration and gD protein expression, suggesting that there is a mechanism to inhibit the activation of Rap1b.

The PBR and CAAX regions at the carboxyl end of Rap1b participate in its phosphorylation (inactivation) and prenylation (activation) modifications, and there is a relationship between the two modifications that is competitive and mutually inhibitory [22,45]. The modification of prenylation is the premise of Rap1b activation, which endows Rap1b with a stronger affinity for the membrane and promotes subsequent Rap1b membrane localization and functions after GTP loading under the action of GEFs, thus participating in regulating various signal pathways [22]. To understand whether the activation manner of Rap1b is caused by post-translation modification, the Rap1b mutants, as shown in a schematic diagram displayed in Figure 2D, were constructed and designated as Rap1b (wild-type), Rap1b (AA) (phosphodeficient mutant), Rap1b (EE) (phosphomimetic mutant), and Rap1b-CAAX (prenylation mutant). Encouragingly, transient expression of the full prenylation mutant (Rap1b-CAAX) accelerated virus infection in a highly significant dose-dependent manner, even higher by about 33% at a 0.5 μg transfection amount than the Rap1b (AA) mutant with a 3 μg transfection amount, which broke the limit of wild-type Rap1b’s promoting effect on virus infection at a high transfection dose (Figure 2E). More significantly, the fully phosphorylated mutant (Rap1b(EE)) no longer promoted viral infection, and the phosphodeficient mutant (Rap1b(AA)) with a higher probability of prenylation also showed an advantage over wild-type Rap1b in promoting virus infection at each transfection amount, especially the higher one. 

### 3.3. Dephosphorylation of PKA Is Conducive to HSV-1 Infection

**Figure 3:**
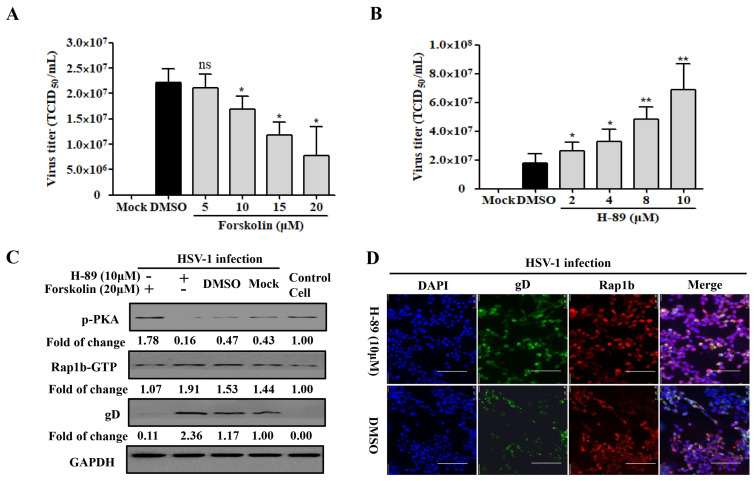
PKA is responsible for HSV-1 infection involving Rap1b activation. (**A**,**B**) PKA activity regulates HSV-1 infection. HCE cells were pretreated with different concentrations of Forskolin, H-89, or DMSO at 37 °C for 1 h, then infected with HSV-1 (MOI = 2) for 24 h in the presence of drugs. Viral titers were measured using TCID_50_. Mock-treated cells served as the blank control. Data represent the results from three independent experiments in triplicate (Mean ± SD; * *p* < 0.05; ** *p* < 0.01; ns, not significant). (**C**) PKA dephosphorization promotes Rap1b activation and induces HSV-1 infection. HCE cells were pretreated with Forskolin (20 μM), H-89 (10 μM), or DMSO for 1 h, then infected with HSV-1 for 24 h in the presence of drugs. Viral titers were measured using TCID_50_, and viral gD and total Rap1b proteins were detected using Western blotting. GAPDH was used as an internal loading control. Mock-treated cells served as the blank control. Cell treatment with PKA inhibitor H-89 (10 μM) and HSV-1 (MOI = 2) infection was visualized with IFA for Rap1b and viral gD proteins. DMSO-treated cells served as the negative control. Scale bar: 100 μm (**D**).

The phosphorylation of the upstream limiting factor PKA in Rap1b is connected to the membrane-remodeling-related physiological process [46]. By recruiting the PKA agonists forskolin and H-89 in HSV-1-infected cells, a desired decline and increase in virus infection were both detected in a dose-dependent manner (Figure 3A,B). Consistently, the PKA agonist forskolin counteracted HSV-1 infection-induced PKA dephosphorylation and significantly restored PKA phosphorylation with less Rap1b activation and viral gD protein (Figure 3C). Conversely, dephosphorylation of PKA concomitant with increased Rap1b activation and viral gD protein was prominently reversed with the participation of PKA inhibitor H-89 (Figure 3C), which was further proved through visual differences using confocal microscopy (Figure 3D).

### 3.4. HSV-1 IE Gene ICP4 Promotes Virus Infection by Inhibiting PKA Activation

**Figure 4:**
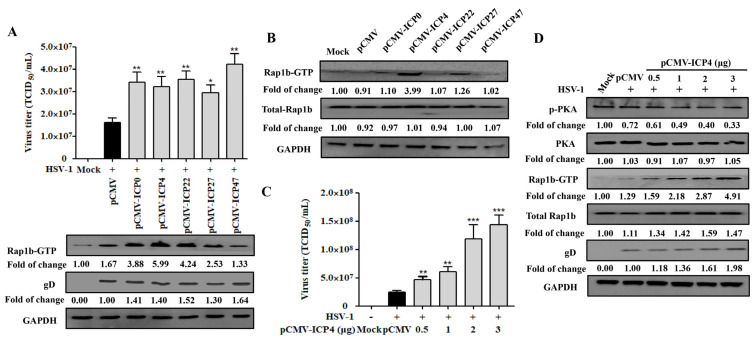
HSV-1 IE gene ICP4 drives Rap1b activation by inhibiting PKA phosphorylation. (**A**) Transient transfection of five HSV-1 IE genes promotes varying degrees of Rap1b activation and HSV-1 infection. HCE cells were transfected with 1 μg of pCMV-ICP0, pCMV-ICP4, pCMV-ICP22, pCMV-ICP27, pCMV-ICP47, or negative control plasmid (pCMV) for 24 h, then infected with HSV-1 (MOI = 2) for another 24 h. Viral titers were measured using TCID_50_, and the detection of viral gD and total Rap1b proteins using Western blotting. GAPDH was used as an internal loading control. Mock-treated cells served as blank control. Data represent the results from three independent experiments in triplicate (Mean ± SD, * *p* < 0.05, ** *p* < 0.01). (**B**) Transient transfection of five recombinant plasmids of IE genes promotes Rap1b activation to varying degrees. HCE cells were transfected with 1 μg of pCMV-ICP0, pCMV-ICP4, pCMV-ICP22, pCMV-ICP27, pCMV-ICP47, or negative control plasmid (pCMV) for 24 h, and then the total Rap1b and Rap1b-GTP protein expression was measured using Western blotting. GAPDH was used as an internal loading control. Mock-treated cells served as the blank control. (**C**) Transient transfection of ICP4 plasmid promotes HSV-1 infection in a dose-dependent manner. HCE cells were transfected with different concentrations of pCMV-ICP4 or negative control plasmid (pCMV) for 24 h, then infected with HSV-1 (MOI = 2) for another 24 h. Viral titers were measured using TCID_50_, and the detection of viral gD and total Rap1b proteins was conducted using Western blotting. GAPDH was used as an internal loading control. Mock-treated cells were used as the blank control (Mean ± SD, ** *p* < 0.01, *** *p* < 0.001). (**D**) Transient transfection of ICP4 plasmid promotes Rap1b activation and HSV-1 infection in a dose-dependent manner by inhibiting PKA phosphorylation. HCE cells were transfected with different concentrations of pCMV-ICP4 or negative control plasmid (pCMV) for 24 h, then infected with HSV-1 (MOI = 2) for another 24 h. Protein expression levels of p-PKA, PKA, Rap1b-GTP, total Rap1b, and viral gD were measured using Western blotting. GAPDH was used as an internal loading control. Mock-treated cells served as the blank control.

Due to the activation characteristics of Rap1b-GTP increase and membrane enrichment since the early stage of HSV-1 infection, we proposed the spontaneous hypothesis that the achievement of Rap1b activation was associated with HSV-1 IE genes. As shown in Figure 4A, the correlation between Rap1b activation and a specific IE gene has not yet been obtained, and all five IE genes exhibited the ability to promote viral infection. Subsequent data from cells transfected with different IE-gene-related plasmids without virus infection suggested that ICP4, and perhaps ICP27, showed a significant role in promoting the activation of Rap1b (Rap1b-GTP) without HSV-1 infection, which may allow the virus to infect through Rap1b activation more efficiently (Figure 4B). In addition, further experiments with different amounts of the ICP4 plasmid also showed dose–dependent promotion of HSV-1 infection, as shown above (Figure 4C). More importantly, with the increase in ICP4 plasmid transfection, PKA dephosphorylation and Rap1b activation showed a significant increase, accompanied by more gD protein and total Rap1b expression, when compared with HSV-1-infected cells transfected with the control plasmid (pCMV) (Figure 4D). Overall, these observations suggest that the HSV-1 IE gene ICP4 may dominate or partially promote Rap1b activation during virus infection through the inhibition of PKA phosphorylation.

### 3.5. Rap1b Activation Regulated CDC42 Cascade Signaling Pathway Participates in Viral Infection through Cell Membrane Remodeling

**Figure 5:**
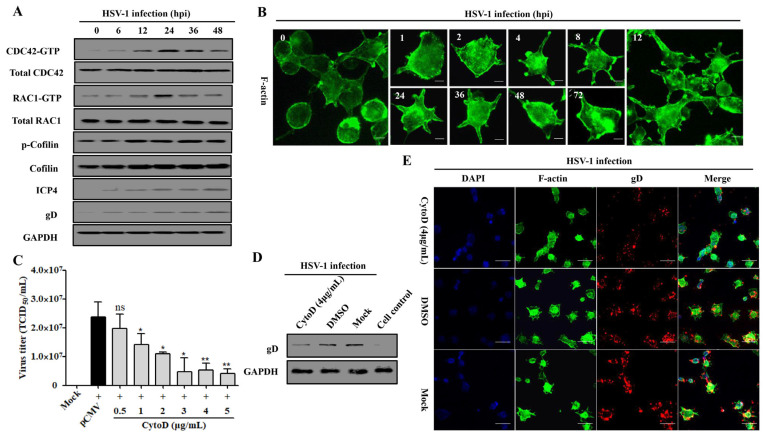
CDC42- and RAC1-stimulated membrane remodeling is involved in HSV-1 infection. (**A**) HSV-1 infection induces the activation of the CDC42-RAC1-Cofilin signaling pathway. HCE cells were infected with HSV-1 (MOI = 2). Cells were lysed at indicated time points to detect the CDC42-RAC1-Cofilin activation pathway and viral ICP4 and gD proteins using Western blotting. GAPDH was used as an internal loading control. (**B**) HSV-1 infection promotes a dynamic change in membrane remodeling. HCE cells were infected with HSV-1 (MOI = 2) and fixed at different time points for membrane remodeling using F-actin staining. Scale bar: 5 μm (**C**) F-actin inhibitor CytoD inhibits HSV-1 infection in a dose-dependent manner. HCE cells were pretreated with different concentrations of CytoD or DMSO at 37 °C for 1 h, then infected with HSV-1 (MOI = 2) for 24 h. Viral titers were measured using TCID_50_. Data represent the results from three independent experiments in triplicate (Mean ± SD, * *p* < 0.05, ** *p* < 0.01, ns, not significant). Viral gD protein levels in HSV-1 infected cells treated with CytoD (4 μg/mL) or DMSO were measured using Western blotting. Mock-treated and control cells served as the negative and blank controls, respectively (**D**); cells treated as in C were fixed for IFA visualization of membrane remodeling and virus infection using F-actin and viral gD protein staining. Mock-treated cells served as the negative control (**E**).

The Rho family proteins CDC42 and RAC1 are participants in confusing mechanisms during HSV-1 infection [37,47], but our previous work has elucidated their causal relationship in response to Rap1b activation in membrane remodeling [26,31]. Here, the Cofilin protein, a direct executor downstream of CDC42 and RAC1 for the conversion of actin monomer (G-actin) and polymer (F-actin), was strongly activated with the activation of CDC42 and RAC1 in a time-dependent manner [26,31,48] during the whole process of HSV-1 infection, which was accompanied by the increased expression of ICP4 and gD proteins (Figure 5A). As a result, Cofilin mediated skeleton-based membrane remodeling with dynamic change that was visually observed at different points after infection (Figure 5B), which is consistent with the activation of the cascade signal pathway (Figure 5A). Consistent with the above results, treatment with CytoD, an F-actin inhibitor that restricts its depolymerization [49], also inhibited virus infection in a dose-dependent manner (Figure 5C). Furthermore, the virus infection decreased with attenuated cell remodeling under the action of an inhibitor (Figure 5D,E).

## 4. Discussion

**Figure 6:**
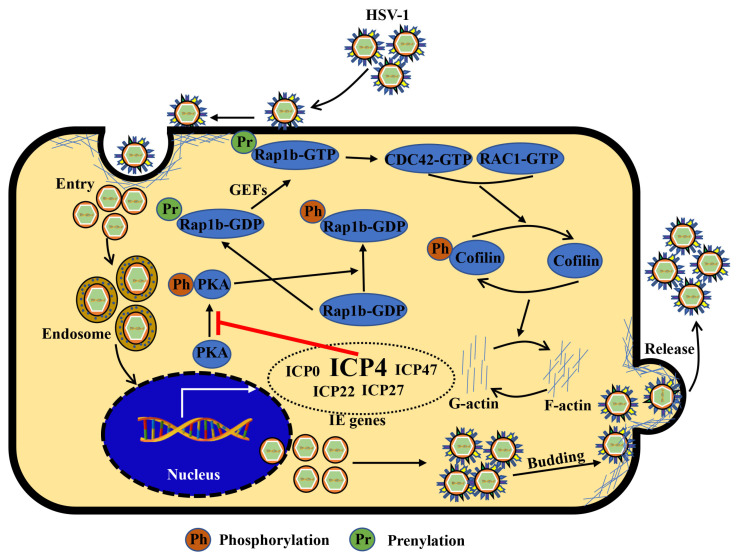
Schematic diagram: ICP4 triggers Rap1b-CDC42-RAC1-Cofilin cascade signaling for membrane remodeling during HSV-1 infection. Viral entry starts with membrane invagination mediated by membrane receptors. In the cytoplasm, the viral genome, with the lipid capsule and capsid removed, was transported and released into the nucleus for genomic transcription and translation, especially the IE genes. Among them, ICP4 products may affect other IE gene functions supernumerary of PKA activation inhibitors, resulting in the overactivation of Rap1b and CDC42-RAC1-Cofilin cascade signaling pathways, the frequent remodeling of the cell membrane, which is beneficial for all membrane-associated processes during HSV-1 infection, including entry, intracellular transport and release (budding), etc., and finally, may provide a favorable microenvironment for efficient viral infection.

HSV-1 infection has a significant worldwide prevalence, but addressing this issue remains neglected due to its non-fatal consequences [10,50]. However, recently, increased mortality in severe COVID-19 patients has been strongly associated with HSV-1 reactivation [51]. Notably, other studies also suggest HSV-1 co-infection with other viruses, such as human immunodeficiency virus (HIV) [52], SARS-CoV-2 [53], Epstein–Barr virus (EBV) [54], and adeno-associated virus 2 (AAV2) [55], can promote symptom recurrence and worsen the infection. Therefore, understanding the mechanism of HSV-1 infection in facial epithelial cells is key to controlling latent infection in the primary sites and preventing potential disease progression. In the current study, we showed that the host Rap1b was involved in F-actin-mediated membrane remodeling following HSV-1 infection in HCE cells by targeting the CDC42-Cofilin cascade and that this is driven by HSV-1 IE ICP4 (Figure 6). These results indicate an important role for Rap1b during HSV-1 infection, which is likely not limited to epithelial cells given Rap1b’s broad tissue distribution (https://www.proteinatlas.org/search/Rap1b (accessed on 23 March 2022)). For these reasons, Rap1b may be a potential target for host-directed therapy against HSV-1 infection that requires further investigation.

Amino acid composition change in the PBR region of Rap1b directly determines its affinity with the cell membrane, which is achieved through the competition of posttranslational modifications between phosphorylation or prenylation [22] and acts as a membrane recruiting partner for subsequent cytoskeleton function initiation [31]; however, the full molecular mechanism remains to be determined, especially during virus infection. Rap1b activation and membrane accumulation emerged simultaneously during HSV-1 infection, similar to previous reports of HEV-induced Rap1b activation [26]. However, Rap1b activation seems to occur throughout the entire process of HSV-1 infection, while in HEV infection, it is restricted to early infection events (i.e., virus attachment or penetration process), which may result from the intrinsic properties of the experimental models, as cell lines only support early entry HEV with inefficient replication [26,56], while for HSV-1 and many other viruses, cell lines allow for efficient replication and cell-to-cell transmission [28,29,40,57]. Rap1b activation is likely to play a role in all processes related to the cell membrane because of its extremely close correlation with the cytoskeleton [58], as proved by the impact on cytoskeleton rearrangement following Rap1b siRNA, plasmids, or drug inhibitors currently in HSV-1 infection evaluation. Additionally, the evaluation of progeny virus production during the whole investigation period does not indicate whether Rap1b is involved in virus ingress, intracellular replication, or egress yet. Future studies should use these tools to unravel the role of Rap1B in different stages of viral replication.

Electrostatically mediated interactions between prenylated Rap1b with activation potential on the cell membrane [22] and chaperone proteins, including cyclase-associated protein 1 (CAP1) [45], smg GDP dissociation stimulator (SmgGDS) [59], and protease-activated receptor (PAR) [60], lead to efficient GTP-loading and activation (Rap1b-GTP) through PKA [26,46]. However, this study detected no dose-dependent increase in virus infection or Rap1b activation with a gradient increase in Rap1b expression in the HSV-1-infected cells. Nonetheless, treating cells with phosphorylation and prenylation-interrelated mutants before virus infection significantly improved and promoted the activation of Rap1b and viral infection in a dose-dependent manner, indicating that competition between phosphorylation and prenylation modification in the PBR of Rap1b may be the main factors limiting the activation of Rap1b during HSV-1 infection rather than GTP loading from the limitation of GEFs [22]. Furthermore, dephosphorylation of PKA during HSV-1 infection was induced, and the employment of PKA agonists and inhibitors affected virus infection by inhibiting and promoting Rap1b activation with a negative correlation, respectively. Hence, how HSV-1 inhibits PKA activation to initiate Rap1b activation remains unknown and requires further study.

Previous studies have reported that five IE genes of HSV-1 participate in self-genome expression and host interference regulation during the HSV-1 early infection stages, supported by the multifunctional domains of the ubiquitin ligase E3 region from ICP0 [61], the N- and C-terminal catalytic region and DNA binding region from ICP4 [13], the core conserved region from ICP22 [62], the N-terminal half region from ICP27 [63], and the unique short (US) region from ICP47 [64], respectively. Rap1b activation is significantly up-regulated at 6 hpi after HSV-1 infection, followed by recruitment to the cell membrane, suggesting that IE genes of HSV-1 may be involved. However, all five HSV-1 IE genes promoted virus infection to different degrees in overexpressing cell lines, with a similar impact on Rap1b activation, which could not clarify whether Rap1b activation was driven by IE genes or a signaling cascade reaction initiated by rapid virus infection. Interestingly, subsequent experiments using different IE overexpression plasmids without HSV-1 infection showed that ICP4 had the highest Rap1b activation level. However, total Rap1b protein expression was not upregulated in IE transfected cells without virus infection, implying that Rap1b translation is driven by separate mechanisms [65]. Still, virus infection increased in a dose-dependent manner under conditions of increasing ICP4-induced PKA dephosphorylation. Thus, the likely crosstalk between HSV-1 IE gene ICP4 and PKA-driven host Rap1b protein transfer for Rap1b-pushed membrane motion was probed and found to have a causal relationship, which needs to be checked further with IE gene knockout viruses in the future.

Membrane-remodeling-based cell morphological changes contribute to many physiological processes. Viruses can hijack these processes to facilitate infection, especially during ingress and egress [25,66]. Here, similar remodeling was observed, using a confocal microscope, in HSV-1 infection of HCE cells after staining F-actin with phalloidin. Its activity was characterized by the frequent formation of stress fibers, lamellipodia, and filopodia [25,26]. These results showed consistency with previously described work during early HSV-1 infection [37,47,67] and were also observed previously with HEV [26,31], PHEV [25], porcine reproductive and respiratory syndrome virus (PRRSV) [68], and HIV [69]. Furthermore, the inhibition of cell remodeling with the treatment of the F-actin inhibitor CytoD blocked the emergence of virions on the cell surface during early HSV-1 infection, indicating a positive correlation between F-actin, remodeling, endocytosis, and virus entry [70,71]. However, specific molecular mechanisms of membrane regulation during the early HSV-1 infection period remain elusive. Interestingly, the functions of Cofilin-F-actin dynamics are likely diverse in the whole HSV-1 infection process, including entry [71], intracellular transport and replication [39], and egress [66], due to the broad-spectrum roles of the membrane for cross-linking among actin, tubulin, or non-muscle myosin II (NMII) motor proteins, etc., to produce mechanical force and tracks [72]. In addition, a strong connection between CDC42 cascade signal activation and HSV-1 infection was validated, which was in agreement with previous studies [67,73] but conflicted with the conclusion that RAC1 and CDC42 are not essential for HSV-1 infection in keratinocytes [47], suggesting a mechanism discrepancy among different cell types. 

## 5. Conclusions

We have shown a significant correlation between Rap1b activation and HSV-1 infection in HCE cells. We highlight the potential of Rap1b as a molecular switch determining membrane-mediated HSV-1 infection. Thereupon, Rap1b, CDC42 cascade signaling, and Cofilin, the direct executor of cytoskeleton regulation, are likely to be potential host therapeutic targets for antiviral strategies during the early and lytic infection of HSV-1 in epithelial cells. 

## Figures and Tables

**Table 1:** Primer pairs used in this study for the construction of recombinant plasmids.

Primer Name	Sequences (5′–3′)
Rap1b-F	GGAATTCATGCGTGAGTATAAGCTAGTCGTTCTTGGC
Rap1b-R	CGCTCGAGAAGCAGCTGACATGATGACTTT
Rap1b(AA)-F	GAATTCATGCGTGAGATAAAGCTAGTCGTTCTTGGCTCAGGAGGCGTTGG
Rap1b(AA)-R	GCTCGAGAAGCAGCTGACA**AGCAGC**CTTTTTGCGAGCCTTCCCA
Rap1b(EE)-F	CCGGAATTCATGCGTGAGTATAAGCTAGTCGTTCTTGGCTAAGGAGGCGTT
Rap1b(EE)-R	CCGCTCGAGAAGCAGCTGACA**CTCCTC**CTTTTTGCGAGCCTTCCC
Rap1b-CAAX-F	CCGGAATTCATGCGTGAGTATAAGCTAGTCGTTCTTGGCTCAGGAGGCGT
Rap1b-CAAX-R	CCGCTCGAGAAGCGAATG**TGATGATGA**CTTTTTGCGAGCCTTCCC
ICP0-F	GGAATTCATGGAGCCCCGCCCCGGAG
ICP0-R	GCTCTAGATTGTTTTCCCTCGTCCCGGGTCGACGC
ICP4-F	GGAATTCATGGCGTCGGAGAACAAGCAGCGC
ICP4-R	GCTCTAGACAGCACCCCGTCCCCCTCGAAC
ICP22-F	GGAATTCATGGCCGACATTTCCCCAGGCG
ICP22-R	GCTCTAGACGGCCGGACCAACGTGTCGCTG
ICP27-F	GGAATTCATGGCGACTGACATTGAT
ICP27-R	GCTCTAGAAAACAGGGAGTTGCAATAAAAATATTTGC
ICP47-F	GGAATTCATGTCAACGGGTTACCGGATT
ICP47-R	GCTCTAGAATGTCGTGGGCCCTGGAA

Underline and bold font represent the digestion site and mutation site, respectively.

**Table 2:** Target siRNA sequences used in this study.

siRNA	Sequences (5′–3′)
siRap1b(sense)	CCACAUUUAACGAUUUACATT
siRap1b(antisense)	UUCUGUUAAUUUGCCGCACTT
siRNA(sense)-negative control	UUCUCCGAACGUGUCACGUTT
siRNA(antisense)-negative control	ACGUGACACGUUCGGAGAATT

## Data Availability

The data used to support the findings of this study are available from the corresponding author upon request.
